# Genome-Wide Identification of the *IDD* Gene Family in Soybean (*Glycine max*) and Their Expression Profiles in Response to Drought, Salt Stress, and Different Photoperiod Conditions

**DOI:** 10.3390/genes17040489

**Published:** 2026-04-20

**Authors:** Rouxing Li, Zixiang Ning, Zhihui Dong, Jian Xi, Chenjie Shi, Xianlian Chen, Qingyuan He, Shaochuang Chuang, Xue Yang, Yingjie Shu

**Affiliations:** 1 College of Agriculture, Anhui Science and Technology University, Chuzhou 233100, China; LRX675789@outlook.com (R.L.); ningzixiang@ahstu.edu.cn (Z.N.); D200004@outlook.com (Z.D.); 15156119935@163.com (J.X.); scj1610881167@outlook.com (C.S.); xlchen5200@163.com (X.C.); 2 College of Life and Health Sciences, Anhui Science and Technology University, Chuzhou 233100, China; heqy@ahstu.edu.cn (Q.H.); chuangsc@ahstu.edu.cn (S.C.); 3 Anhui Province Key Laboratory of Functional Agriculture and Functional Food, Anhui Science and Technology University, Chuzhou 233100, China

**Keywords:** *Glycine max*, *IDD* gene family, abiotic stress, photoperiod, gene expression

## Abstract

**Background:** INDETERMINATE DOMAIN proteins (IDDs) are a plant-specific transcription factor family, and members of this family play crucial roles in regulating growth and development as well as environmental adaptation. However, a comprehensive analysis of the *IDD* family in soybean [*Glycine max* (L.) Merrill] is limited. **Methods and Results:** A total of 27 *GmIDD* genes were identified in the soybean genome, unevenly distributed across 14 chromosomes, and their encoded proteins all harbor a conserved INDETERMINATE (ID) domain with two Cys2His2 (C2H2) and two Cys2HisCys (C2HC) zinc finger motifs. Phylogenetic analysis classified these *GmIDD* genes into three subgroups. Soybean *GmIDD* genes exhibit high homology with their *Arabidopsis thaliana* IDD counterparts. Cis-acting element analysis indicated that the promoters of *GmIDD* genes are enriched in light-responsive elements (such as Box4), hormone-responsive elements (such as ABRE and AuxRR-core), and abiotic stress-responsive elements (such as MBS and LTR). The qRT-PCR results showed that *GmIDD3*/*5*/*14*/*22*/*26* were upregulated under salt stress, while *GmIDD8*/*9*/*10*/*12*/*16*/*17*/*19*/*20*/*23*/*24*/*25*/*27* were obviously downregulated during treatment. Under drought stress, the expression levels of *GmIDD4*/*6*/*7*/*10*/*14*/*16*/*19*/*22*/*24*/*25*/*26*/*27* were upregulated during the treatment. The expression levels of *GmIDD1*/*2*/*3*/*4*/*12*/*14*/*15*/*16*/*17*/*18*/*22*/*23*/*25*/*26* were induced by short-day conditions, whereas *GmIDD9*/*13*/*19*/*21* were induced by long-day conditions in soybean leaves. **Conclusions:** This study provides a theoretical basis for further understanding the functions of the soybean IDD gene family in abiotic stress tolerance and photoperiod adaptability.

## 1. Introduction

The precise regulation of plant growth, development, and stress responses relies on complex transcriptional regulatory networks. Among numerous transcription factor families (TFs), the INDETERMINATE DOMAIN (IDD) family has attracted widespread attention due to its pivotal roles in plant-specific biological processes. Members of this family belong to the subfamily of Cys2His2 (C2H2) zinc-finger structure transcription factors, with their most distinctive molecular feature being a highly conserved INDETERMINATE (ID) domain located at the N-terminus of the protein. This domain is typically composed of two C2H2 and two Cys2HisCys (C2HC) zinc-finger motifs [1,2,3]. A growing body of evidence demonstrates that *IDD* genes perform diverse functions in *Arabidopsis*, with key roles also being characterized in major crops such as *Zea mays* (maize) and *Oryza sativa* (rice) [4,5,6,7].

Recent years have witnessed breakthrough advances in the functional studies of *IDD* genes in model plants, gradually unveiling the complexity and multifunctionality of their regulatory networks. In the regulation of growth and development, members of the IDD family have been established as core regulatory factors governing pivotal transitions in the plant life cycle. As a pivotal phase connecting vegetative and reproductive development, flowering time has been clearly shown to be controlled by *IDD* genes in maize, rice and *Arabidopsis*. The first identified *IDD* family gene in maize, *ZmID1*, initiates the transition from vegetative to reproductive growth by indirectly regulating the expression of the mobile florigen signal *ZEA CENTRORADIALIS 8* (*ZCN8*), and its mutant exhibits a delayed- or even non-flowering phenotype [3,8]. ZmID1 regulates ZCN8 not through direct promoter binding, but by activating other downstream transcription factors to mediate signal transduction [9]. In rice, the ZmID1 ortholog INDETERMINATE 1/OsID1 (EHD2/RID1) serves as the master switch for floral transition, directly binding to the TTTGTC motif in the promoters of florigen genes such as *HEADING DATE 3A* (*Hd3A*) and *RICEFLOWERING LOCUS T1* (*RFT1*) to activate their expression and initiate flowering [1,10,11,12]. Moreover, *OsIDD1*, *OsIDD4* and *OsIDD6* exhibit functional redundancy with *OsID1*, as overexpression of any one of these genes can restore flowering in the non-flowering *OsID1* mutant [12]. In *Arabidopsis*, NUTCRACKER/AtIDD8 (NUC) participates in the regulation of photoperiodic flowering by directly binding to the conserved CTTTTGTCC motif in the promoter of the *SUS4* gene, thereby modulating sucrose metabolism [13]. Concurrently, its transcriptional activation activity can be inhibited through phosphorylation by the SUCROSE NONFERMENTING-1-RELATED PROTEIN KINASE 1 (SnRK1) kinase [14].

In terms of environmental stress responses, *IDD* genes have been confirmed as key regulators enabling plants to cope with various abiotic stresses. In response to temperature stress, the *Arabidopsis AtIDD14* gene undergoes alternative splicing to produce two isoforms, α and β. Under low temperatures, AtIDD14β forms non-functional heterodimers with AtIDD14α, which inhibits the binding and regulation of downstream starch metabolism genes such as *Qua-QuineStarch* (*QQS*), thereby reducing starch degradation during cold stress [15]. The *SHOOT GRAVITROPISM5*/*AtIDD15* (*SGR5*) gene generates two alternative splicing variants: the full-length SGR5α and a truncated SGR5β isoform lacking functional ZF motifs. Under high-temperature conditions, the production of the truncated SGR5β is specifically induced. This variant functions by suppressing the activity of the full-length SGR5α, ultimately leading to a reduction in the gravitropic response of inflorescence stems [16]. In rice, OsIDD3 (ROC1) activates the expression of the cold-responsive gene *CBF1* and its downstream target *CBF3* by directly binding to the *CBF1* promoter region, and its mutant exhibits a cold-sensitive phenotype [17]. In response to drought stress, AtIDD14 directly interacts with the ABRE-binding factor 1-4 (ABF1-4) transcription factors within the ABA signaling pathway, enhancing their transcriptional activation activity to regulate stomatal closure and water loss rate [18]. Moreover, *IDD* genes are also involved in saline–alkaline stress responses. In rice, OsIDD10 interacts with the brassinazole-resistant 1 (BZR1) protein to activate the expression of *AMT1;2*, enhancing ammonium uptake in roots and consequently improving plant tolerance to saline–alkaline stress [19]. In gravitropic responses, AtIDD15 affects gravity perception and stem circumnutation by regulating the accumulation and sedimentation rate of starch granules in shoot tissues [20,21,22]. In rice, the *LOOSE PLANT ARCHITECTURE 1*/*OsIDD14* (*LPA1*), a homologous gene of *AtIDD15*, modulates shoot gravitropism and plant architecture by influencing amyloplast sedimentation through regulating the adaxial growth of the tiller node and lamina joint [23].

Within hormone signaling transduction and photoperiod regulatory networks, IDD genes function as pivotal nodal proteins, enabling the crosstalk and integration of multiple signaling pathways. In the gibberellin (GA) signaling pathway, AtIDD3, AtIDD4, AtIDD5, AtIDD9 and AtIDD10 can interact with the DELLA protein REPRESSOR OF GA1-3 1 (RGA1) to cooperatively activate the transcription of *SCARECROW-LIKE3* (*SCL3*), a positive regulator of GA signaling, thereby forming a feedback regulatory loop for GA signals [24]. Furthermore, GAI-ASSOCIATED FACTOR1/AtIDD2(GAF1) can bind to DELLA proteins to form a complex that regulates the expression of GA biosynthesis genes such as *GA20ox2* [25,26,27,28]. In the auxin signaling pathway, AtIDD14, AtIDD15, and AtIDD16 promote auxin biosynthesis and polar transport by directly binding to the promoters of genes such as *TRYPTOPHAN AMINOTRANSFERASE of ARABIDOPSIS1* (*TAA1*), *PINFORMED1* (*PIN1*), and *YUCCA5* (*YUC5*) [22]. OsIDD3 indirectly influences plant resistance to sheath blight by repressing the expression of the auxin transporter gene *PIN-FORMED 1b* (*PIN1b*) [29,30]. In the brassinosteroid (BR) signaling pathway, OsIDD3 negatively regulates plant resistance to sheath blight by activating the expression of genes such as *D2*, *D11* and *BRI1* [31].

Soybean is a globally crucial oilseed crop and protein source, and its developmental processes are highly susceptible to environmental factors. Both abiotic stresses and changes in photoperiod can significantly impact flowering time, plant architecture establishment, and yield formation in soybean [32]. Previous studies have shown that *GmIDD*, a member of the *IDD* gene family in soybean, is induced by short days in soybean leaves and regulated by the circadian clock. Under long-day conditions, GmIDD suppresses the expression of *AGAMOUS-like 18* (*AGL18*) by binding to its promoter, thereby inducing the expression of *FLOWERING LOCUS T* (*FT*) and promoting flowering in *Arabidopsis* [33]. However, systematic genome-wide identification, evolutionary analysis, and expression profiling under different photoperiods and abiotic stresses are still lacking.

In this study, we performed genome-wide systematic identification and bioinformatic characterization of the *IDD* family genes in soybean. A comprehensive analysis was performed to determine its member size, phylogenetic relationships, gene structures, conserved motifs, and promoter cis-acting elements. Furthermore, the expression patterns of *IDD* genes in soybean under drought, salt stress, and different photoperiod conditions (long-day/short-day) were investigated. This research aims to elucidate the molecular characteristics and potential environmental adaptation functions of *IDD* genes in soybean, thereby providing new genetic resources and a theoretical foundation for breeding stress-tolerant soybean varieties with improved photoperiod adaptability.

## 2. Materials and Methods

### 2.1. Identification and Characteristics of IDD Members in Soybean

To achieve genome-wide identification of the *IDD* gene family, the complete genome sequences and corresponding annotation data for soybean (Version 6.1), rice and *Arabidopsis* were retrieved from the Phytozome v13 database (https://phytozome-next.jgi.doe.gov/ (accessed on 3 November 2024)). Members of the soybean *IDD* gene family were systematically identified using a combined approach based on the Hidden Markov Model (HMM) and local BLAST (Basic Local Alignment Search Tool, Version 2.11.0). The soybean proteome was searched using BLAST (E-value ≤ 1 × 10^−5^) with a query dataset of *Arabidopsis* (16 members) and rice (14 members) IDD protein sequences to identify potential homologs. The Zn-C2H2 domain file (PF00096) from the Pfam database (http://pfam-legacy.xfam.org/ (accessed on 3 November 2024)) was downloaded and uploaded to the HMMERv3.3.2 (https://www.ebi.ac.uk/Tools/hmmer/ (accessed on 3 November 2024)) to search for potential genes containing this conserved domain in the soybean genome, with an E-value < 1 × 10^−5^ [34]. The candidate sets from both approaches were merged and redundant entries were removed. For gene loci with multiple transcript isoforms, the longest coding sequence (CDS) and its corresponding protein sequence were selected for subsequent analyses to minimize potential interference from alternative splicing. Potential pseudogenes were excluded based on the presence of disrupted reading frames and lack of transcriptional support. All the remaining candidate genes were subjected to domain architecture analysis using NCBI Batch CD-Search (https://www.ncbi.nlm.nih.gov/Structure/cdd/wrpsb.cgi (accessed on 3 November 2024)). Only proteins harboring the full INDETERMINATE (ID) domain were retained as candidate IDD members. This domain is characterized by two Cys2His2 (C2H2) and two Cys2HisCys (C2HC) zinc finger motifs arranged in a conserved manner at the N-terminus. Thus, the 27 *GmIDD* genes identified represent the set of canonical, functional IDD family members in the soybean genome. Protein physicochemical properties, such as molecular weight (MW), isoelectric point (pI), and grand average of hydropathy (GRAVY), were analyzed using ExPASy (Version 3.0, https://www.expasy.org/ (accessed on 3 November 2024)). The subcellular localization of IDD family proteins was predicted using PSORT (Version II, https://www.genscript.com/psort.html (accessed on 3 November 2024)).

### 2.2. Chromosomal Location

The chromosomal position data of the *IDD* genes were obtained and visualized using TBtools-II v2.376 software. According to the order of gene location on the chromosome, they were named *GmIDD1*-*GmIDD27*, respectively. The corresponding relationship between each *GmIDD* gene and its official gene locus identifier, chromosome coordinates, and the closest *Arabidopsis IDD* homologous gene are listed in Supplementary Table S1.

### 2.3. Multiple Sequence Alignment and Phylogenetic Tree Analysis of IDD Proteins

The GmIDD sequences were aligned using Clustal X2.0 software with default parameters and the Multiple Alignment Mode. The alignment results were analyzed for conserved regions in GeneDoc Ver 2.7.000, with underlines for annotation. Multiple sequence alignment of a total of 79 IDD protein sequences from soybean (27 members), *Arabidopsis* (16 members), rice (15 members) and maize (22 members) was performed using Clustal X2.0. Based on the alignment results, a phylogenetic tree of the IDD protein family members was reconstructed using the MEGA11 and the maximum likelihood method, with the bootstrap value set to 1000 replicates. Online tool iTOL (Version 6, https://itol.embl.de/ (accessed on 10 January 2025)) was used to visualize the phylogenetic tree.

### 2.4. Gene Structure and Protein Motif Analysis

To analyze the gene structure, the intron–exon organization was determined using the “Visualize Gene Structure (Basic Version)” feature in TBtools-II v2.376 software. Conserved motifs of the full-length IDD protein sequences were predicted using MEME (Version 5.5.8, http://meme-suite.org/ (accessed on 15 January 2025)), with the number of motifs set to 10. The prediction results were subsequently visualized using TBtools-II v2.376. Batch CD-search (https://www.ncbi.nlm.nih.gov/Structure/bwrpsb/bwrpsb.cgi (accessed on 15 January 2025)) was used to analyze the conserved domain structure.

### 2.5. Analysis of Duplication and Collinearity

We employed the “One Step MCScanX”, “MCScanX File Merge,” and “Dual Synteny Plot” functions of TBtools-II v2.376 to calculate synteny among homologous *IDD* genes both within and between species. The results, including syntenic relationships across *Arabidopsis*, rice, maize and *soybean*, were subsequently visualized.

### 2.6. Cis-Acting Element Analysis of the GmIDD Genes

We extracted the 2000-bp promoter sequences upstream of the initiation codon (ATG) for all *GmIDD* genes using TBtools-II v2.376 and submitted them to the PlantCARE (http://bioinformatics.psb.ugent.be/webtools/plantcare/html/ (accessed on 15 January 2025)) for predicting cis-acting regulatory elements. The cis-acting elements in the promoter regions were visualized using TBtools-II v2.376 [35].

### 2.7. Plant Materials, Methods of Abiotic Stress and Different Photoperiod Treatments

Seeds of the “Williams 82” cultivar were provided by the Legume Crop Engineering Technology Research Center, Anhui Science and Technology University, Chuzhou, China. They were then surface-sterilized by soaking in 1% sodium hypochlorite (analytical grade, Sinopharm Chemical Reagent Co., Ltd., Shanghai, China) solution for 15 min, followed by three rinses with deionized water. The surface-sterilized seeds were evenly spread on moist filter paper and germinated at 25 °C in a growth chamber for 3 days. Subsequently, the germinated seeds were transferred to 1/2 Hoagland nutrient solution (Coolaber Science & Technology Co., Ltd., Beijing, China) for hydroponic cultivation. The plants were grown under controlled conditions: 25 °C, 250 µmol m^−2^ s^−1^ white light under long-day (16/8 h light/dark) conditions (LDs). NaCl and PEG-6000 (polyethylene glycol, MW 6000) were purchased from Shanghai Macklin Biochemical Co., Ltd. (Shanghai, China). When the first trifoliate leaves were expanded, stress treatments were initiated by supplementing the 1/2 Hoagland nutrient solution with either 150 mmol/L NaCl (salt) or 20% PEG-6000 (drought). The control group was maintained in normal 1/2 Hoagland nutrient solution throughout the experiment. Leaf samples from both the treated and control groups were collected at 0, 1, 3, 6 and 12 h after treatment initiation.

To analyze the expression patterns of all *GmIDD* genes under different photoperiods, seeds of “Williams 82” were planted in a greenhouse at 25 °C with 250 µmol m^−2^ s^−1^ white light under LDs. On day 15 after emergence, a subset of seedlings was transferred to short-day (8/16 h light/dark) conditions (SDs) for separate LD and SD treatments. Prior to transfer, all plants were grown under LDs for 15 days to ensure uniform growth and development. After 15 days of growth under the respective photoperiod conditions, leaf samples were collected at seven time points (ZT0, 4, 8, 12, 16, 20 and 24h), with lights-on defined as ZT0. This sampling design, with frequent time points across a full light–dark cycle, allowed the capture of both diurnal expression patterns and potential phase shifts in gene expression induced by the change in photoperiod. All samples were frozen in liquid nitrogen and stored at −80 °C.

### 2.8. Quantitative RT-PCR Analysis

RNA isolation has been described previously [36]. qRT-PCR amplifications were performed using the 2 × SYBR Green qPCR Mix (BOLAZ, Nanjing, China) according to the manufacturer’s instructions, on an ABIViiA7 real-time PCR instrument (Life Technologies, Carlsbad, CA, USA). The PCR cycling conditions were as follows: 95 °C for 15 min; 40 cycles of 95 °C for 10 s, 60 °C for 20 s, and 72 °C for 30 s. Gene-specific primers for *GmIDD* genes were designed using Primer3 (https://bioinfo.ut.ee/ (accessed 10 February 2025)). Primer specificity was checked with NCBI Primer-BLAST and further confirmed by a single peak in the melting curve analysis. *GmActin4* (GenBank accession number AF049106) was used as a reference gene [37,38,39] (Table S2). The experiment was conducted with three biological replicates and three technical replicates. The relative expression level of each gene was calculated using the 2^−∆∆Ct^ method, and the data are presented as the average values of three biological replicates ± standard deviation (SD). SPSS software (Version 20) was used for the statistical analysis. A two-tailed Student’s *t*-test was performed to compare each treatment time point (1, 3, 6, 12 h) independently against the 0 h control. Significance was determined at * *p* < 0.05, ** *p* < 0.01. This repeated pairwise testing without multiple correction is a simplified statistical approach.

## 3. Results

### 3.1. Identification of Members of the Soybean IDD Gene Family

**Figure 1:**
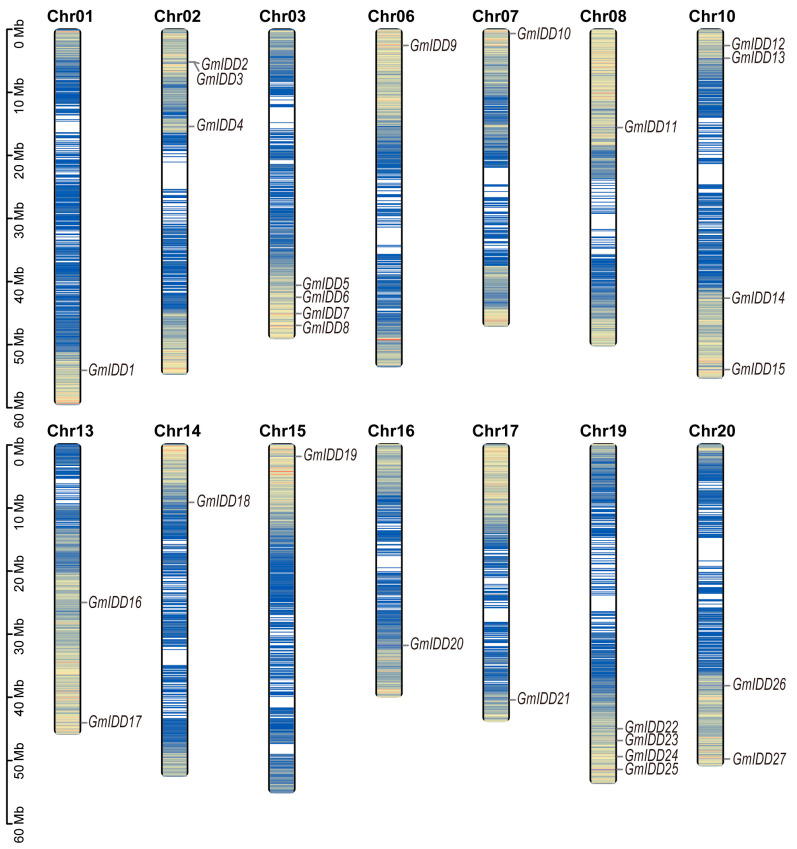
Chromosomal distribution of *IDD* family genes in soybean. The scale bars on the left indicate the length (Mb) of the chromosomes. The blue lines refer to low gene density, and the red lines refer to high gene density. The yellow and orange lines represent intermediate gene density levels (yellow: medium-low; orange: medium-high), and the white background indicates regions with no gene density data or intergenic spaces.

The Hidden Markov Model (HMM) and local BLAST (Basic Local Alignment Search Tool) were combined to systematically identify the members of the soybean *IDD* gene family. After merging candidates and removing duplicates, we selected the longest transcript for each gene locus. Pseudogenes were excluded. Domain analysis with NCBI Batch CD-Search kept only proteins containing the complete INDETERMINATE (ID) domain (two C2H2 and two C2HC motifs). A total of 27 *IDD* family genes were finally screened and identified in soybean. To understand the distribution characteristics of the *GmIDD* genes on the chromosomes, we analyzed the chromosomal location of the *GmIDD* genes using TBtools. The results showed that the 27 *GmIDD* genes were unevenly distributed on fourteen chromosomes (Figure 1). These *IDD* genes were named according to their position on the chromosome, in the order of *GmIDD1* to *GmIDD27*. Chromosomes 03, 10 and 19 each harbored four gene members. Chromosome 02 carried three gene members, while chromosomes 13 and 20 each had two gene members. All remaining chromosomes contained only one gene member. Furthermore, *GmIDD2* and *GmIDD3* formed a tight gene cluster on chromosome 02 (Figure 1).

The amino acid sequence characteristics of the GmIDD family proteins indicated that the number of amino acids in GmIDD proteins ranged from 408 (GmIDD18) to 571 (GmIDD15), and their molecular weights were between 45.1 and 61.7 kDa. The theoretical isoelectric point (pI) ranged from 7.51 to 9.4. The instability index of GmIDD proteins ranged from 38.72 (GmIDD4) to 61.19 (GmIDD1), and the aliphatic index ranged from 48.78 (GmIDD26) to 71.21 (GmIDD18). The grand average of hydropathicity (GRAVY) values for all GmIDD proteins were negative (Table 1), indicating an overall hydrophilic tendency at the whole-protein level. However, as with many transcription factors, this does not preclude the existence of local hydrophobic regions, which may be essential for structural integrity or DNA binding. Subcellular localization predictions consistently targeted all GmIDD proteins to the nucleus (Table 1), consistent with their predicted function as transcription factors, although experimental validation is still needed to confirm these predictions.

### 3.2. Gene Structure and Protein Motifs of IDD Genes

**Figure 2:**
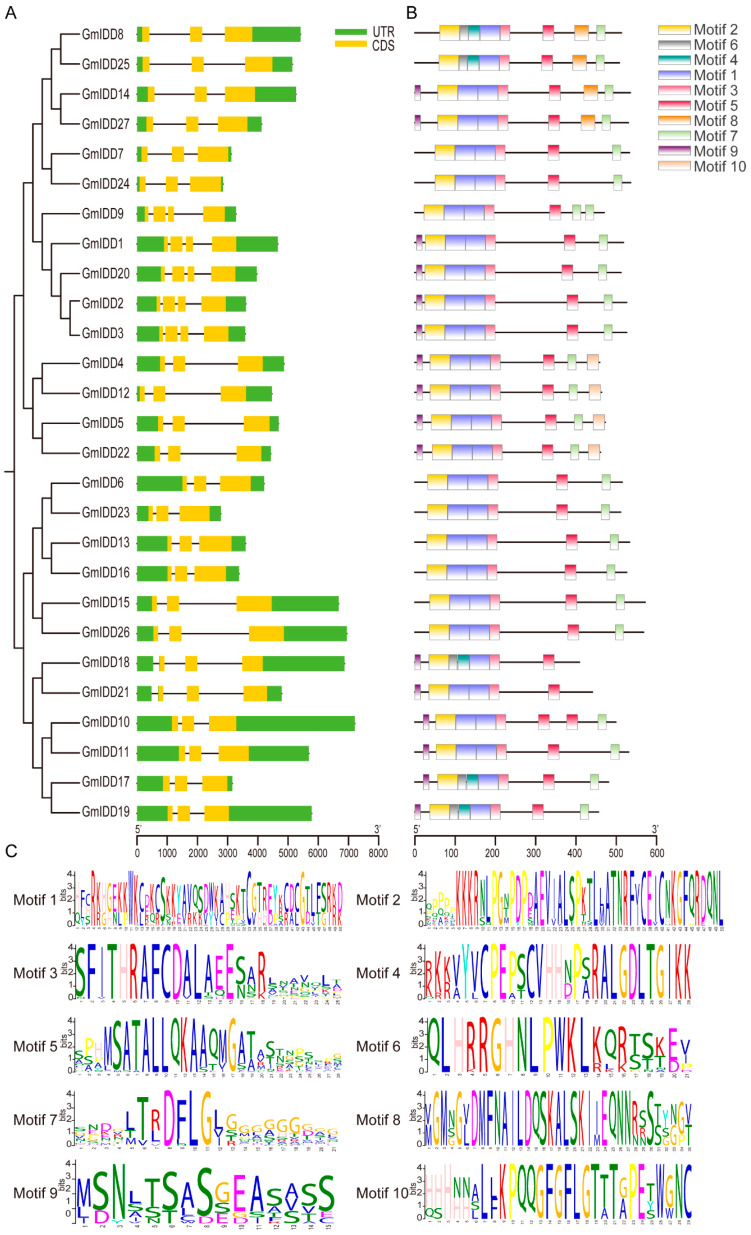
Conserved motifs and gene structure of 27 *IDD* family members in soybean. (**A**) Exon/intron structures of *GmIDD* genes. UTR, untranslated region; CDS, coding sequence. (**B**) Conserved motifs of GmIDD proteins. (**C**) The sequence logo conserved motif of GmIDD proteins. The letters represent amino acid residues, and their heights indicate the relative conservation level at each position.

To comprehensively understand the genetic structure, we conducted a detailed analysis of the exon–intron structure of *GmIDD* genes (Figure 2A). The results showed that *GmIDD* genes contained 3 or 4 exons and 2 or 3 introns. Among them, *GmIDD1*/*2*/*3*/*9*/*20* contained 4 exons and 3 introns. In addition to the aforementioned genes, the gene structures of all other genes consisted of 3 exons and 2 introns.

To further explore the motif regions of the IDD family proteins, we analyzed the distribution of motifs in the IDD proteins using the MEME website and predicted 10 conserved motifs (Figure 2B). The lengths of the 10 conserved motifs range from 15 to 50 amino acids (Figure 2C). All GmIDD proteins contained Motif 1, Motif 2, Motif 3 and Motif 5, indicating that these motifs were highly conserved within the IDD family. Except for GmIDD18 and GmIDD21, all other GmIDD proteins contained Motif7, and GmIDD9 had two more of them. Motif8 was present in the closely related GmIDD8/14/25/27. Similarly, GmIDD4/5/12/22 specifically contained Motif10 (Figure 2B).

**Figure 3:**
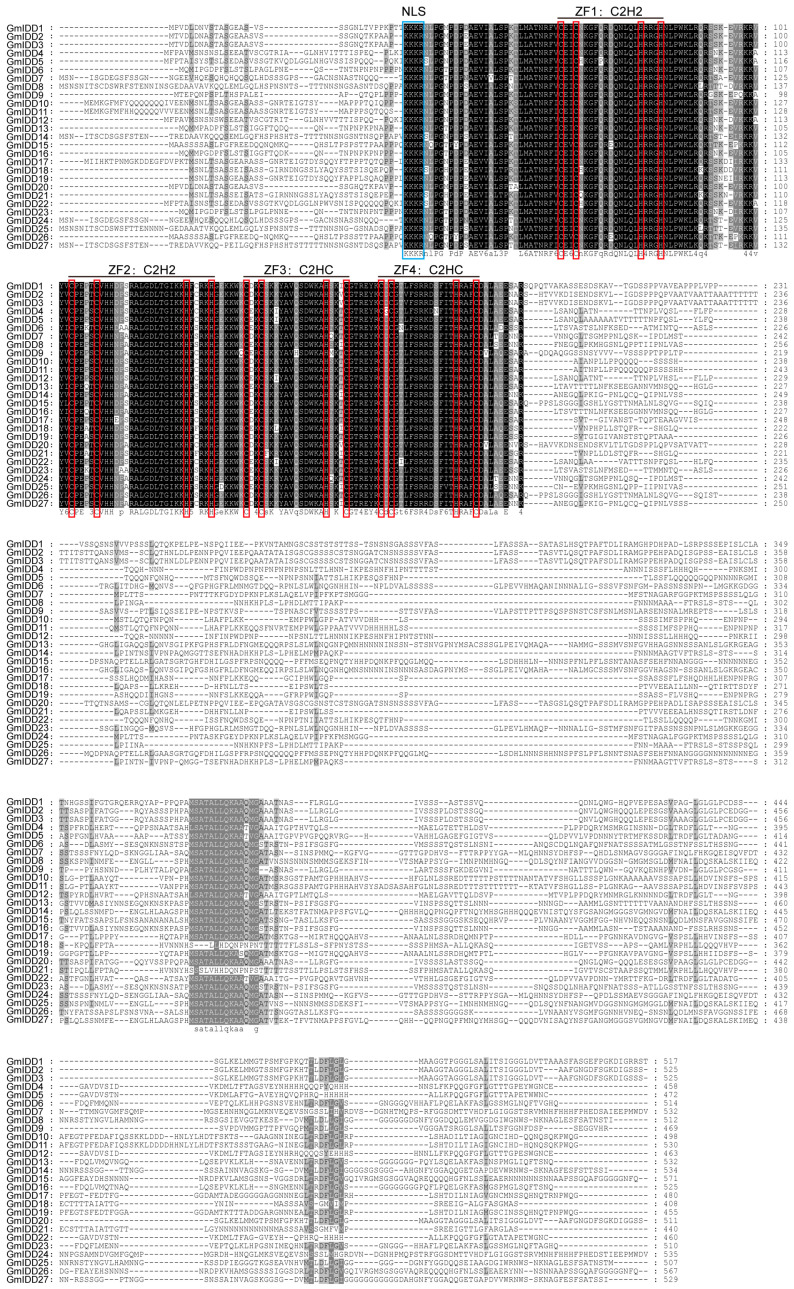
Alignment of INDETERMINATE (ID) domain conserved amino acid sequence in soybean. The blue boxes indicate nuclear localization signal region (NLS). The red boxes indicate zinc finger domains (C, H residues). Black, homology 100%; dark grey, homology ≥75%; light grey, homology ≥50%.

The protein sequences of GmIDD proteins were compared multiple times using Clustal X2.0 (Figure 3), and it was found that the N-terminus of all GmIDD proteins possessed one nuclear localization signal (NLS, KKKR sequence) and a highly conserved ID domain (2 C2H2 and 2 C2HC domains). Sequence alignment further confirmed that Motif 1, Motif 2 and Motif 3 were the major components constituting the conserved ID domain.

### 3.3. Phylogenetic Analysis of IDD Genes

**Figure 4:**
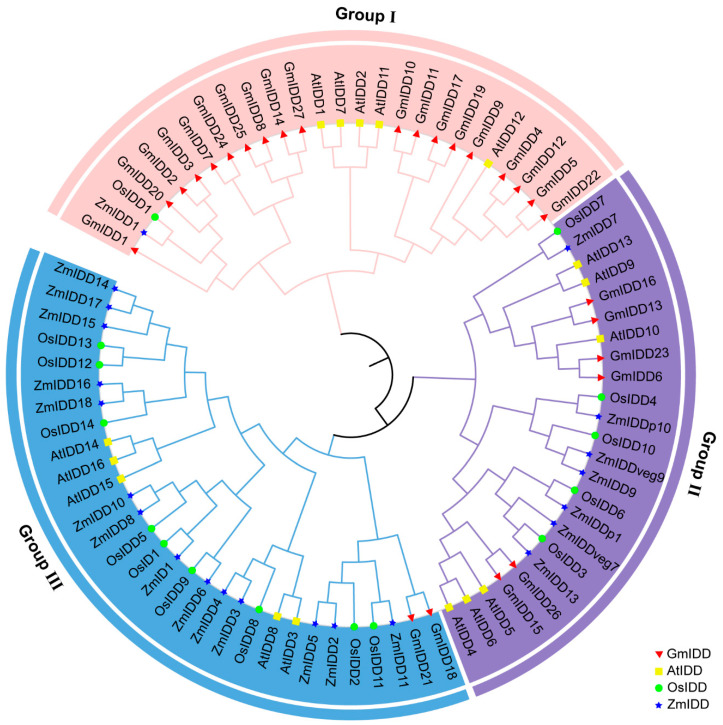
Phylogenetic tree of IDD family proteins from soybean (Gm), *Arabidopsis* (At), rice (Os) and maize (Zm).

To investigate the phylogenetic relationship of *GmIDD* family genes, a phylogenetic tree of *IDD* genes was constructed using Clustal X2.0 sequence alignment and MEGA11 software (Maximum Likelihood method, ML), including four species: soybean (27 members), rice(15 members), *Arabidopsis*(16 members) and maize (22 members) (Table S3). Based on the results of the phylogenetic tree and referring to the IDD family classification standards of *Arabidopsis* and rice, the 27 GmIDD proteins were divided into three subgroups: I, II and III (Figure 4). In subgroups I, II and III, there were 2, 19 and 6 members of the GmIDD protein, respectively.

**Figure 5:**
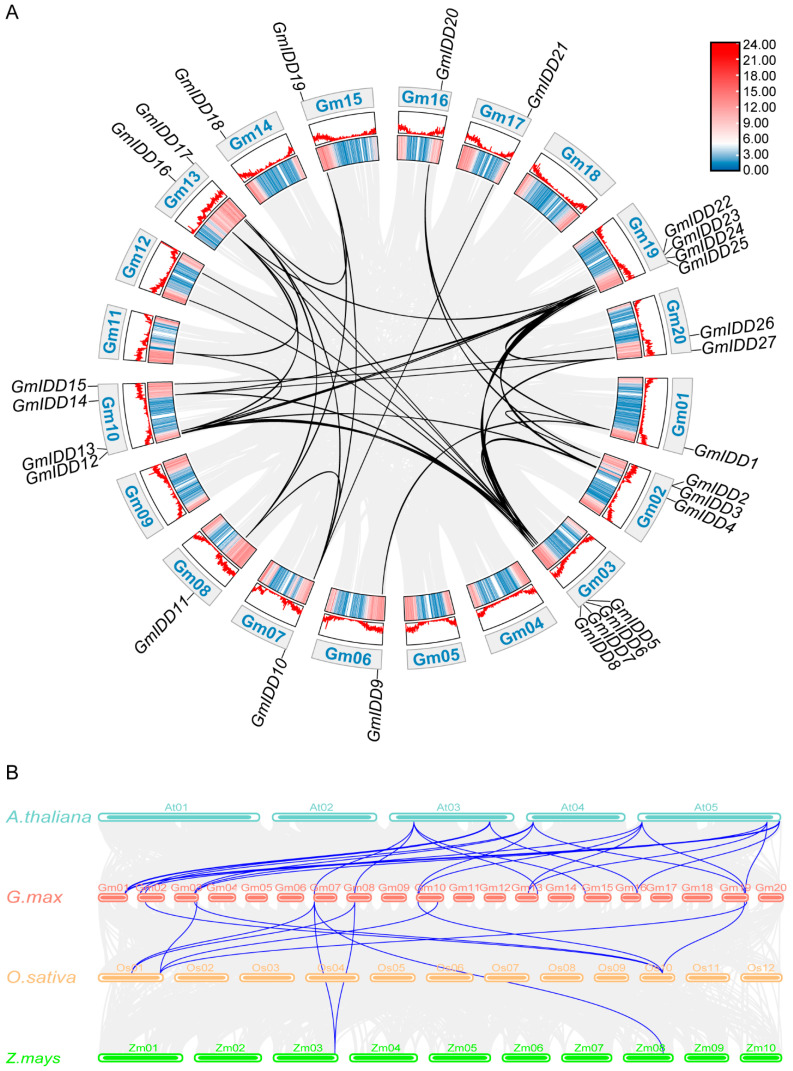
Collinearity analysis within and between species of *IDD* family genes in soybean. (**A**) Collinearity analysis of *IDD* family genes in soybean. Gray lines indicate all synteny blocks in the soybean genome. The genes linked by black lines represent homologues. (**B**) Collinearity analysis between *IDD* genes in soybean (*G. max*), *Arabidopsis* (*A. thaliana*), rice (*O. sativa*) and maize (*Z. mays*). The gray line represented all genes with synteny between the two species, and the blue lines highlight syntenic *IDD* gene pairs.

By using MCScanX and MicroSynteny to analyze the duplication relationships of the *GmIDD* genes, it was found that among the 27 *GmIDD* genes, there were 35 pairs of segmental duplication gene pairs (Figure 5A). Synteny analysis between soybean and the other three species was used to study the evolutionary relationship of *IDD* family genes (Figure 5B). The results showed that all the soybean *IDD* genes had a syntenic relationship with 21 *IDD* genes in *Arabidopsis*, 9 *IDD* genes in rice and 3 *IDD* genes in maize. This indicated that the *IDD* gene family in soybeans has a higher degree of homology with the *IDD* gene family in *Arabidopsis*, and this result was consistent with the results of the constructed evolutionary tree.

### 3.4. Distribution of Cis-Acting Elements on the Promoter of GmIDD Genes

**Figure 6:**
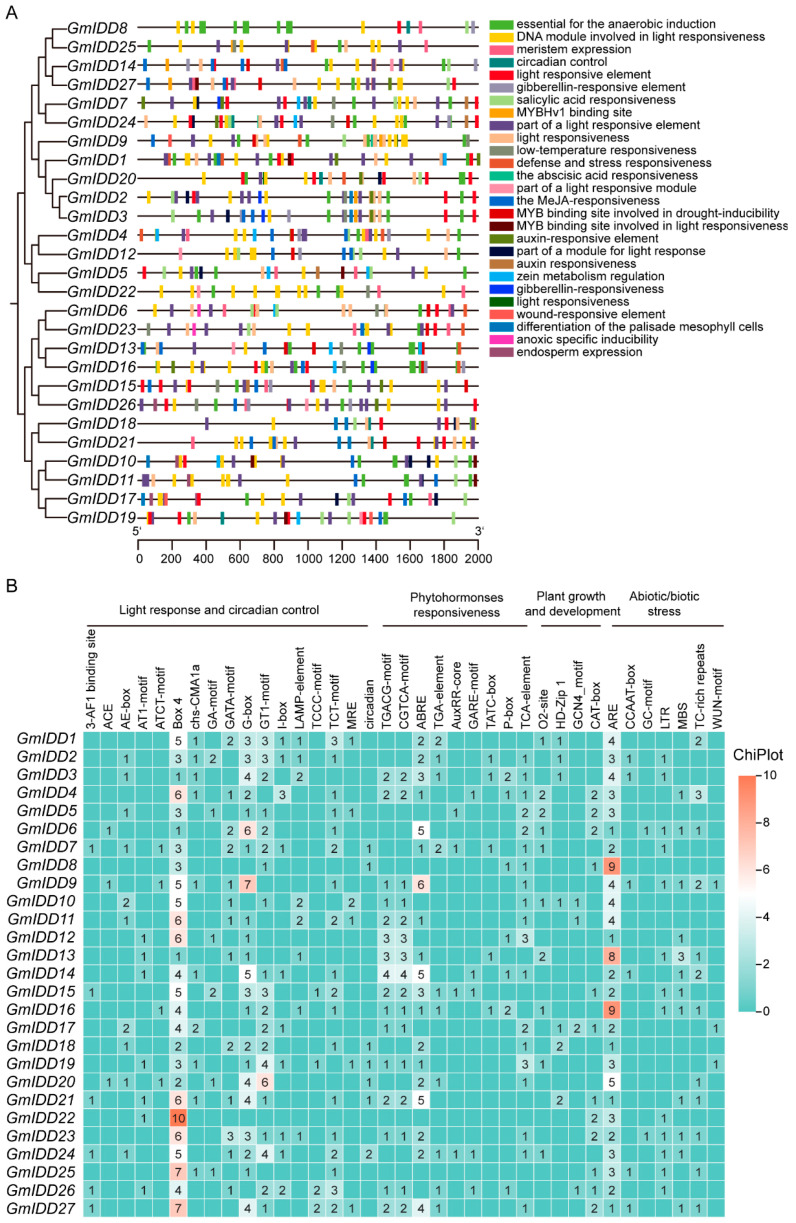
Analysis of cis-acting elements of *IDD* family promoters in soybean. (**A**) Predicted cis-acting elements in *GmIDD* promoters. The bottom scale indicates the direction and length of the sequence. (**B**) The types and numbers of cis-acting elements in the promoter of the *GmIDD* genes.

To investigate the cis-acting elements in the promoter of *GmIDD* genes, the sequence approximately 2000 bp upstream of the translation initiation site was analyzed. After excluding core promoter components such as CAAT-box and TATA-box, the remaining cis-acting elements were classified into four functional groups (Figure 6A,B). The first category was related to abiotic/biotic stress, including drought (MBS, CCAAT-box), low temperature (LTR), anaerobic induction (ARE), wound response (WUN-motif), hypoxia induction (GC-motif), and defense and stress response (TC-rich repeats). The second category was related to plant growth and development elements, including regulatory elements involved in zein metabolism regulation (O2-site), endosperm expression (GCN4-motif), differentiation of palisade mesophyll cells (HD-ZIP1) and meristem expression (CAT-box). The third category encompasses elements linked to phytohormones, such as gibberellins (GARE-motif, P-box, TATC-box), auxins (AuxRR-core, TGA-element), abolic acid (ABRE), salicylic acid (TCA-element) and methyl jasmonate (TGACG-motif, CGTCA-motif). The fourth functional cluster comprised light-responsive and circadian control elements. Among the various components, the number of light-response components ranked first. This suggested that these genes might respond to changes in light intensity or photoperiod, or be involved in photomorphogenesis. In particular, the promoter regions of all *GmIDD* genes contained 1 to 10 copies of Box4, *GmIDD3* contained 1, and *GmIDD22* contained 10.

### 3.5. Expression Pattern Analysis of GmIDDs Under Drought and Salt Stress Treatments

When the first trifoliate leaf of soybean seedlings was fully expanded, the seedlings were subjected to drought (20% PEG-6000) and salt (150 mmol/L NaCl) stress treatments. Leaf samples were collected at 0, 1, 3, 6 and 12 h after the initiation of treatment. Expression differences in *GmIDDs* under drought and salt treatments were analyzed by qRT-PCR. The results indicated that there were certain differences in the expression patterns of each gene under different treatments.

**Figure 7:**
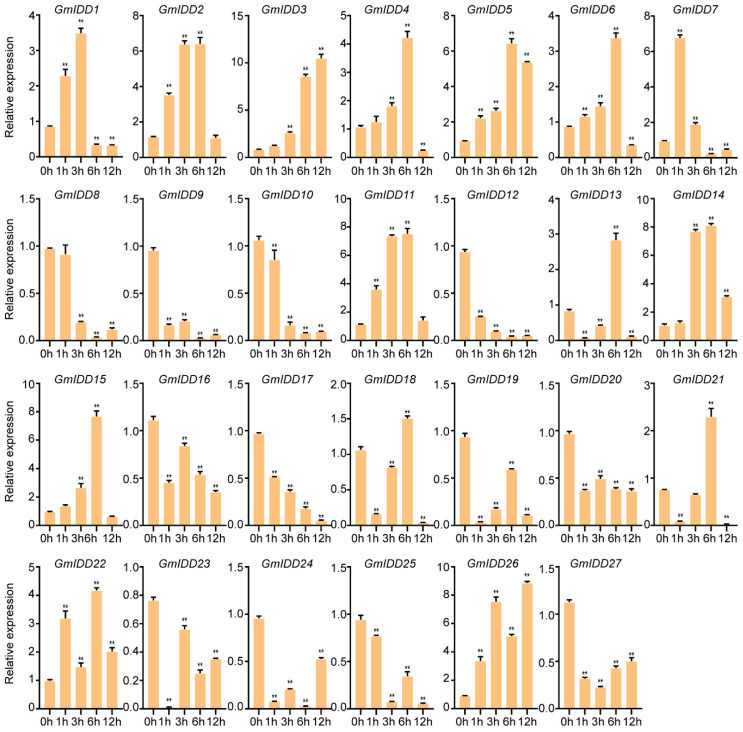
Expression pattern analysis of the *GmIDD* genes under salt (150 mmol/L NaCl) stress. Values are presented as means ± SD (*n* = 3). The asterisks on the top of the columns indicate significant differences from the value at 0 h (** *p* < 0.01, Student’s *t*-test).

Under salt stress (150 mM NaCl), the expression levels of *GmIDD3*/*5*/*14*/*22*/*26* were upregulated throughout the treatment period. *GmIDD2* and *GmIDD11* showed progressive upregulation from 1 to 6 h, and then dropped back to the original level at 12 h. In contrast, *GmIDD8*/*9*/*10*/*12*/*16*/*17*/*19*/*20*/*23*/*24*/*25*/*27* were obviously downregulated during treatment. Several genes exhibited a biphasic expression pattern: *GmIDD1* and *GmIDD7* were downregulated early during treatment (1–3 h) and then significantly upregulated at 6–12 h. *GmIDD4*/*6*/*15* reached their peak expression at 6 h after treatment, followed by a slight decline at 12 h. *GmIDD13*/*18*/*21* showed a transient upregulation only at 6 h post-treatment, while their expression remained significantly downregulated at other time points (Figure 7).

**Figure 8:**
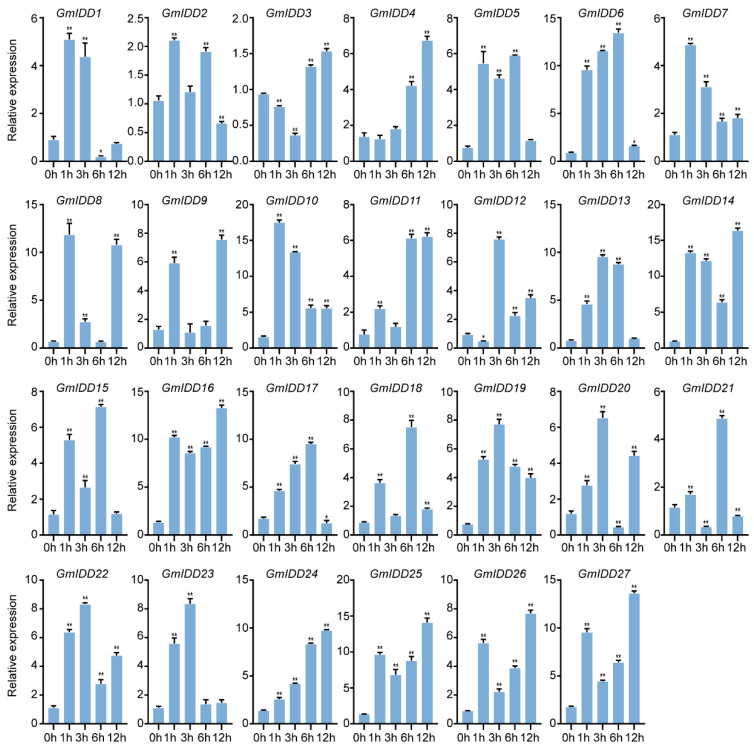
Expression pattern analysis of the *GmIDD* genes under drought (20% PEG-6000) stress. Values are presented as means ± SD (*n* = 3). The asterisks on the top of the columns indicate significant differences from the value at 0 h (* *p* < 0.05, ** *p* < 0.01, Student’s *t*-test).

Under drought stress, the expression levels of twelve *GmIDD* genes (*GmIDD4*/*6*/*7*/*10*/*14*/*16*/*19*/*22*/*24*/*25*/*26*/*27*) were upregulated throughout the treatment period. *GmIDD5*/*13*/*15*/*17* exhibited an initial upregulation at 1 to 6 h followed by a decline at 12 h. *GmIDD1* and *GmIDD23* showed transient induction, reaching peak expression at 1 h or 3 h and then declining during 6 to 12 h. *GmIDD3* and *GmIDD12* showed a trend of first decreasing temporarily and then increasing. Five genes (*GmIDD2*/*8*/*9*/*20*/*21*) exhibited irregular fluctuations (Figure 8).

### 3.6. Expression Pattern Analysis of GmIDDs Under Different Photoperiod Conditions

**Figure 9:**
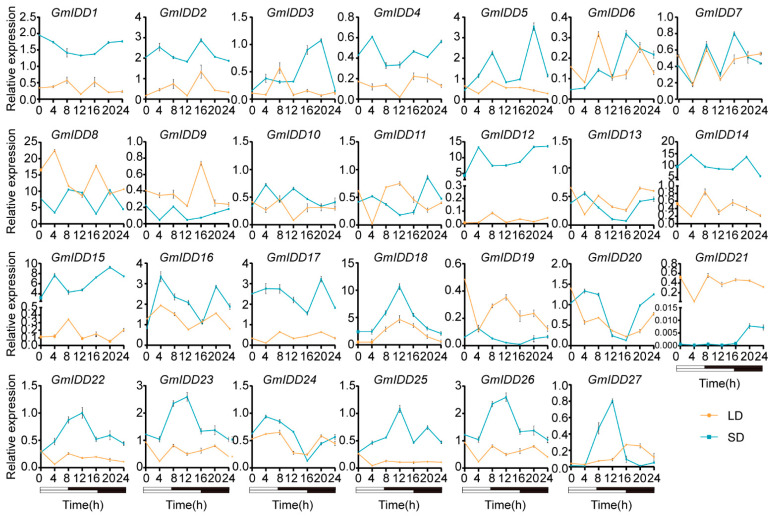
Expression pattern analysis of the *GmIDD* genes under different photoperiod conditions. White and black bars at the bottom represent light and dark phases, respectively. LD and SD respectively represent long-day (16/8 h light/dark) and short-day (8/16 h light/dark) conditions. Values are presented as means ± SD (*n* = 3).

To investigate the photoperiodic responsiveness of the *GmIDD* gene family, we analyzed their expression patterns under long-day (16/8 h light/dark) conditions (LDs) and short-day (8/16 h light/dark) conditions (SDs) at seven time points (0, 4, 8, 12, 16, 20 and 24 h). The results showed that the expression levels of *GmIDD1*/*2*/*4*/*12*/*14*/*15*/*17*/*18*/*22*/*23*/*25*/*26* were consistently higher under SDs than under LDs at all time points. In addition, *GmIDD3* and *GmIDD16* exhibited lower expression under SDs than under LDs at 8 h and 16 h, respectively, while their expression levels were higher under SDs than under LDs at the other time points. Therefore, the aforementioned 14 genes were induced by SDs in soybean leaves. Conversely, *GmIDD9* and *GmIDD21* displayed consistently higher expression under LDs than under SDs at all time points. *GmIDD13* and *GmIDD19* showed lower expression under LDs than under SDs at 4 h, but higher expression at all other time points, suggesting that *GmIDD9*/*13*/*19*/*21* genes were induced by LDs (Figure 9).

## 4. Discussion

Soybean is one of the world’s most vital oil and protein crops, and its growth, development and environmental adaptability directly govern yield stability. The *INDETERMINATE DOMAIN* (*IDD*) gene family represents a class of plant-specific C2H2-type zinc finger transcription factors, and has been shown to participate in multiple critical biological processes including growth and development regulation, hormone signal transduction, and abiotic stress responses in species such as *Arabidopsis*, rice and maize. However, systematic characterization and functions of the IDD family in soybean are still absent. In this study, a total of 27 *GmIDD* members were identified at the genome-wide level in soybean, and comprehensive analyses were performed on their phylogenetic evolution, conserved motifs, and cis-acting elements within promoters, as well as expression patterns under diverse photoperiodic conditions and abiotic stresses. These findings lay a solid foundation for further elucidating the biological functions and molecular regulatory networks of *GmIDD* genes in soybean.

The members of the *IDD* gene family in soybean are unevenly distributed across chromosomes (Figure 1). Most of these members contain 3–4 exons, and the N-terminus of their encoded proteins all possess one nuclear localization signal (NLS, KKKR sequence) and a highly conserved ID domain (2 C2H2 and 2 C2HC domains) (Figure 2 and Figure 3). This structural feature is highly consistent with that of IDD proteins in species including *Arabidopsis*, rice and maize [2,40]. Phylogenetic analysis revealed that the 27 GmIDD proteins could be classified into three subgroups (I, II, III), with subgroup II harboring the largest number of members (19) (Figure 4). Synteny analysis of *GmIDD* genes against their orthologs in *Arabidopsis*, rice and maize demonstrated that soybean *IDD* genes shared a closer homologous relationship with those in *Arabidopsis*, suggesting a degree of evolutionary relatedness between the two lineages (Figure 5). This finding also reflects the evolutionary divergence in the *IDD* gene family between soybean, a representative leguminous species, and monocotyledonous Poaceae crops. Cis-acting element analysis revealed that the promoter regions of *GmIDD* genes are enriched with light-responsive elements (Box4), hormone-responsive elements (ABRE, AuxRR-core, GARE-motif), and abiotic stress-responsive elements (MBS, LTR) (Figure 6). Their cis-acting element distribution pattern is similar to that of *AtIDD* gene promoters in *Arabidopsis* [41], implying that soybean IDD may integrate light, hormone, and stress signals to participate in the regulation of complex physiological processes.

Abiotic stress represents a major environmental factor constraining soybean yield, and IDD proteins serve as key regulators in plant stress adaptation [17,18]. In this study, expression analysis under abiotic stress conditions further delineated the potential roles of *GmIDD* genes in plant stress adaptation. The expression levels of *GmIDD3*/*5*/*14*/*22*/*26* were upregulated, whereas those of *GmIDD8*/*9*/*10*/*12*/*16*/*17*/*19*/*20*/*23*/*24*/*25*/*27* were downregulated under salt treatment (150 mM NaCI) (Figure 7), suggesting that distinct *GmIDD* genes may be differentially involved in the salt stress response. Similar patterns of functional divergence have also been observed in the *IDD* gene families of both *Arabidopsis* and maize. In *Arabidopsis*, *AtIDD4* is induced under salt stress and acts as a negative regulator to exacerbate the salt-sensitive phenotype, while *AtIDD14* potentially mediates a positive regulatory role via the ABA signaling pathway [18,42]. In maize, *ZmIDD8* is transcriptionally upregulated under salt stress to positively regulate salt tolerance, whereas *ZmIDD5* is downregulated to negatively modulate the salt stress response, thus exhibiting a distinct pattern of functional divergence [7]. Under drought treatment, we used 20% PEG-6000 in hydroponic culture, which is an osmotic stress model widely used for the initial screening of stress-responsive genes. However, it does not fully represent field drought. Field drought involves gradual soil water depletion, root hydraulic changes, and integrated hormonal responses. The expression levels of twelve *GmIDD* genes (*GmIDD4*/*6*/*7*/*10*/*14*/*16*/*19*/*22*/*24*/*25*/*26*/*27*) were upregulated during the treatment (Figure 8). Cis-acting element analysis revealed that the numbers of both MBS (drought-responsive elements) and ABRE (ABA-responsive elements) were significantly enriched in the promoters of seven upregulated *GmIDD* genes (*GmIDD4*/*6*/*13*/*14*/*15*/*16*/*24*/*27*) (Figure 6). Previous studies have demonstrated that AtIDD14 regulates ABA-mediated drought tolerance by interacting with ABF1-4 in *Arabidopsis* [18]. Similarly, ZmIDD8 and ZmIDD21 integrate ABA signaling via ABA-responsive elements to modulate drought resistance in maize [7]. Based on the enrichment of ABRE elements in their promoters and their transcriptional responses to drought stress, it is plausible that these seven *GmIDD* genes may be involved in ABA-mediated drought response pathways. However, this speculation requires further experimental validation. Therefore, future research should conduct soil drought experiments to verify whether the candidate *GmIDD* genes have practical significance under agriculturally relevant conditions.

Photoperiod is a key environmental factor regulating flowering and yield formation in soybean, and *IDD* genes have been demonstrated to be involved in photoperiod-mediated flowering regulation in *Arabidopsis* and rice [11,13]. This study found that all *GmIDD* gene promoters contain multiple light-responsive elements (such as Box4 and G-box) (Figure 6), raising the possibility that genes of this family may be involved in the light signaling regulatory network. Furthermore, under different photoperiod conditions, the expression of some *GmIDD* genes showed photoperiod-dependent expression patterns. The expression levels of genes including *GmIDD1*/*2*/*3*/*4*/*12*/*14*/*15*/*16*/*17*/*18*/*22*/*23*/*25*/*26* were significantly upregulated under short-day conditions (SDs), whereas *GmIDD9*/*13*/*19*/*21* exhibited increased expression under long-day conditions (LDs) (Figure 9). In *Arabidopsis*, AtIDD8 binds to the promoter of *SUS4* to regulate sucrose metabolism, thus modulating photoperiodic flowering [13]. In rice, OsID1 acts as a key regulator of flowering and directly activates florigen genes such as *Hd3A* and *RFT1* [11]. The photoperiod-responsive *GmIDD* genes identified in this study provide candidate targets for further elucidating the photoperiod-regulated flowering pathway in soybean. Whether they function through sucrose metabolism or flower-inducing hormone regulatory pathways, as reported for their *Arabidopsis* and rice counterparts, remains to be determined experimentally.

## Figures and Tables

**Table 1:** The characteristics of IDD family proteins in soybean.

Gene	Gene ID	AminoAcid Number/aa	Molecular Weight/Da	Theoretical pI	Instability Index	Aliphatic Index	Grand Average of Hydropathicity	Subcellular Localization
*GmIDD1*	*Glyma.01G176600*	517	54,325.01	8.93	61.19	63.66	−0.425	Nucleus
*GmIDD2*	*Glyma.02G058400*	525	54,441.88	8.08	52.63	66.46	−0.313	Nucleus
*GmIDD3*	*Glyma.02G058500*	525	54,441.88	8.08	52.63	66.46	−0.313	Nucleus
*GmIDD4*	*Glyma.02G144400*	458	50,942.17	9.08	38.72	63.30	−0.711	Nucleus
*GmIDD5*	*Glyma.03G157432*	472	51,840.26	9.34	44.38	63.31	−0.586	Nucleus
*GmIDD6*	*Glyma.03G179700*	514	55,818.28	9.29	42.17	58.50	−0.640	Nucleus
*GmIDD7*	*Glyma.03G211700*	532	58,198.76	8.66	53.83	50.41	−0.736	Nucleus
*GmIDD8*	*Glyma.03G236600*	512	55,375.91	8.65	51.64	62.71	−0.611	Nucleus
*GmIDD9*	*Glyma.06G033300*	469	50,062.38	9.17	57.96	64.07	−0.495	Nucleus
*GmIDD10*	*Glyma.07G009100*	498	54,519.23	8.97	53.67	61.02	−0.628	Nucleus
*GmIDD11*	*Glyma.08G192300*	530	58,109.89	8.96	54.12	58.06	−0.691	Nucleus
*GmIDD12*	*Glyma.10G029700*	463	51,750.22	9.36	40.59	67.02	−0.718	Nucleus
*GmIDD13*	*Glyma.10G051500*	532	57,447.10	9.37	43.32	57.99	−0.660	Nucleus
*GmIDD14*	*Glyma.10G153200*	534	57,532.89	8.95	52.68	53.41	−0.691	Nucleus
*GmIDD15*	*Glyma.10G280000*	571	61,730.73	9.30	51.64	49.47	−0.853	Nucleus
*GmIDD16*	*Glyma.13G139000*	525	56,452.02	9.40	42.88	56.91	−0.663	Nucleus
*GmIDD17*	*Glyma.13G349500*	480	52,482.72	8.95	48.87	58.58	−0.719	Nucleus
*GmIDD18*	*Glyma.14G095900*	408	45,129.00	9.25	54.61	71.27	−0.498	Nucleus
*GmIDD19*	*Glyma.15G024500*	455	49,126.97	9.22	48.51	60.35	−0.631	Nucleus
*GmIDD20*	*Glyma.16G141100*	511	52,998.28	7.51	52.41	66.52	−0.326	Nucleus
*GmIDD21*	*Glyma.17G228000*	440	48,499.59	9.28	50.17	68.95	−0.515	Nucleus
*GmIDD22*	*Glyma.19G159600*	460	50,512.81	9.24	42.76	66.63	−0.566	Nucleus
*GmIDD23*	*Glyma.19G180400*	510	55,322.69	9.21	41.91	58.22	−0.650	Nucleus
*GmIDD24*	*Glyma.19G208900*	535	58,395.89	7.68	58.29	49.96	−0.731	Nucleus
*GmIDD25*	*Glyma.19G234500*	507	54,935.35	8.71	53.05	61.05	−0.641	Nucleus
*GmIDD26*	*Glyma.20G109400*	567	61,169.05	9.23	49.47	48.78	−0.827	Nucleus
*GmIDD27*	*Glyma.20G235100*	529	56,754.21	8.87	56.12	55.18	−0.635	Nucleus

## Data Availability

All data analyzed during this study are included in the Supplementary Information Files.

## Supplementary Materials

The following supporting information can be downloaded at: https://www.mdpi.com/article/10.3390/genes17040489/s1, Table S1: Correspondence of provisional *GmIDD* gene names with official locus identifiers, chromosomal coordinates, and their closest *Arabidopsis thaliana* IDD orthologs; Table S2: The specific sequences of the primers; Table S3: Summary of the *IDD* gene members in *Arabidopsis thaliana*, *Oryza sativa* and *Zea mays*.
